# Immune Checkpoint Inhibition in Non-Melanoma Skin Cancer: A Review of Current Evidence

**DOI:** 10.3389/fonc.2021.734354

**Published:** 2021-12-20

**Authors:** Connor J. Stonesifer, A. Reza Djavid, Joseph M. Grimes, Alexandra E. Khaleel, Yssra S. Soliman, Amanda Maisel-Campbell, Tiffany J. Garcia-Saleem, Larisa J. Geskin, Richard D. Carvajal

**Affiliations:** ^1^ Vagelos College of Physicians and Surgeons, Columbia University, New York, NY, United States; ^2^ Department of Dermatology, Columbia University Irving Medical Center, New York, NY, United States; ^3^ Division of Hematology/Oncology, Department of Medicine, Columbia University Irving Medical Center, New York, NY, United States

**Keywords:** non-melanoma skin cancer (NMSC), immunotherapy, squamous cell carcinoma (SCC), basal cell carcimoma (BCC), merkel cell carcinoma (MCC), immune checkpoint inhibition (ICI)

## Abstract

Immuno-oncology is a rapidly evolving field with growing relevance in the treatment of numerous malignancies. The prior study of immunotherapy in dermatologic oncology has largely focused on cutaneous melanoma. However, recent focus has shifted to the use of immunotherapy to treat non-melanoma skin cancers (NMSCs), such as basal cell carcinoma (BCC), cutaneous squamous cell carcinoma (cSCC), and Merkel cell carcinoma (MCC). NMSCs represent the most ubiquitous cancers globally and, while they have a lower propensity to develop into advanced disease than cutaneous melanoma, their absolute mortality burden has recently surpassed that of melanoma. Patients with advanced NMSC are now benefiting from the successes of immunotherapy, including checkpoint inhibition with anti-CTLA-4 and anti-PD-1 monoclonal antibodies. In this review, we discuss the existing clinical evidence for immunotherapy in the treatment of NMSCs, with an emphasis on checkpoint inhibitor therapies. We highlight key studies in the field and provide up-to-date clinical evidence regarding ongoing clinical trials, as well as future study directions. Our review demonstrates that checkpoint inhibitors are positioned to provide unparalleled results in the previously challenging landscape of advanced NMSC treatment.

## Introduction

Recent advances in the field of immuno-oncology have translated into breakthrough treatments for many solid and hematological malignancies. The study of immunotherapy in dermatologic oncology has largely focused on cutaneous melanoma, a disease that is more likely to metastasize and become life-threatening as compared to other non-melanoma skin cancers (NMSCs) such as basal cell carcinoma (BCC) and cutaneous squamous cell carcinoma (cSCC). Indeed, patients with advanced cutaneous melanoma were some of the first to significantly benefit from studies of checkpoint inhibition with anti-CTLA-4 and anti-PD-1 monoclonal antibodies. Despite a lower propensity to develop into advanced disease, NMSCs still remain a significant burden on the healthcare system (1–11). Indeed, NMSCs are the most prevalent cancer globally and the absolute number of deaths each year attributed to BCCs and cSCCs in the US is now greater than that of melanoma.

Patients with advanced NMSC are now benefiting from the successes of immunotherapy previously observed in melanoma. Like cutaneous melanoma, NMSCs are generally characterized by UV damage, which translates into a high tumor mutational burden (TMB). High TMB is associated with the formation of neoantigens, the putative targets of immune cells that recognize and eradicate neoplastic cells. As such, immunotherapeutic strategies used in the treatment of melanoma that energize the immune system against these numerous tumor antigens, as in the case of checkpoint inhibitors or oncolytic viral immunotherapies, would also be predicted to be effective treatments for NMSCs (7, 12). In some cases, these therapies have demonstrated efficacy and are already being applied in the clinic.

In this review, we will discuss the existing clinical evidence for immunotherapy in the treatment of NMSCs, with an emphasis on checkpoint inhibitor therapies. We also discuss possible reasons for heterogeneity of responses among NMSC, ongoing clinical trials, and future study directions for immunotherapy as a therapeutic approach for NMSC.

## Epidemiology

**Figure 1:**
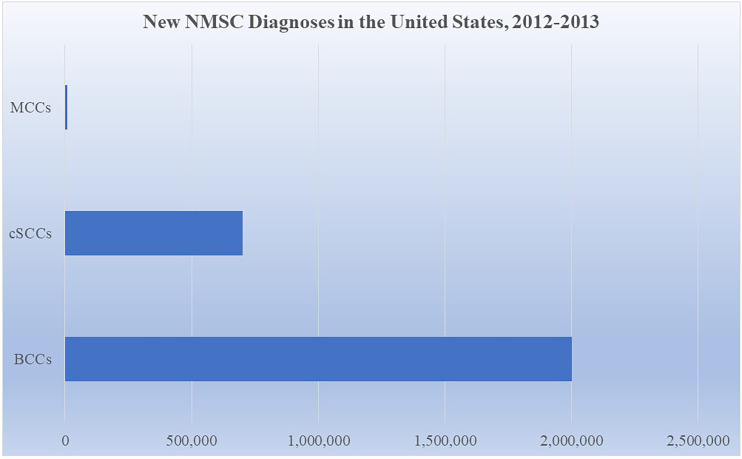
Incidence of new NMSCs in the United States from 2012-2013 (3–5).

NMSCs are the most ubiquitous cancers in the world, estimated to account for over 30% of cancer diagnoses each year (1). However, accurate estimates are limited as many national tumor registries do not routinely assess highly prevalent NMSCs and epidemiologic models frequently fail to consider NMSC incidence in non-white populations. In addition, an assessment of the global burden of disease is challenging due to the need for more numerous population-based studies. While acknowledging the limitations of the epidemiological models available, current studies still point to the significant and growing public health burden NMSCs pose. One model estimates that in the US 5.4 million total NMSCs were diagnosed in 2012 (2). Additional models suggest that 2 million BCCs and 700,000 cSCCs were diagnosed in the US in 2012, whereas 2,488 MCCs were reported in 2013 (see **Figure 1**) (3–5). Globally, the incidence of NMSCs has continued to increase, rising 33% from 2007 to 2017 (1). In the US, the Rochester Epidemiology Project reported a 145% and 263% increase in the incidence of BCCs and cSCCs, respectively, between 1976 to 1984 and 2000 to 2010 (6).

Cumulative UV exposure is considered the chief risk factor in NMSC development (7). Accordingly, the rising global life expectancy and associated increase in total years of UV-exposure are posited as the drivers behind the substantial incidence changes observed. Mortality rates for NMSCs are relatively low, with case fatality rates for cSCCs ranging from 2.1%-2.8% (8, 9). Approximately 4.6% of cSCCs recur after excision and 3.7% progress to nodal metastases (9). However, due to their high prevalence, the absolute mortality from NMSCs remains significant. The absolute number of deaths from cSCCs in 2012 in the US was estimated to range from 3932 to 8791 in the white population alone (4). For comparison, from 2012 to 2016, melanoma absolute deaths in the US across all races and ethnicities was a mean of 9,008 per year, while in 2021 this number has decreased to 7,180 (10, 11) With the incidence of NMSCs predicted to rise at a significant rate, effective therapy is an imperative.

## Immunogenicity

NMSCs represent a class of uniquely immunogenic cancers. In melanoma and other malignancies, TMB and expression of PD-L1 have been demonstrated to correlate with response to checkpoint blockade (12). In non-melanoma cutaneous malignancies, important differences exist in some of these immunological characteristics which may impact their responses to immunotherapy.

### TMB

In 2017, Chalmers et al. published an analysis of the TMB in 92,439 tissue blocks representing over 100 tumor types (see **Table 1** for comparisons) (12). cSCCs and BCCs were found to have the highest TMB of all cancers surveyed, with 45.2 and 47.3 median mutations/Mb, respectively (12). Merkel cell polyomavirus (MCPyV) associated MCCs exhibit a median TMB of only 1.2 mutations/Mb, while non-virus associated MCCs have a high TMB of 53.9 median mutations/Mb (13). The considerably elevated TMB in non-virus associated NMSCs is believed to reflect the chronic carcinogenic effects of ultraviolet light exposure.

**Table 1:** Immune Characteristics of NMSCs (12–20).

	TMB (median mutations/Mb)	PD-L1 expression (Tumor)	PD-L1 expression (TILs)
**BCC**	47.3	22%-89%	82-94%
**cSCC (immunocompetent)**	45.2	25-41%	60%
**MCC (non-virus associated)**	53.9	0%	25%
**MCC (MPyV-associated)**	1.2	50%	56%
**Cutaneous melanoma**	13.5	30%-35%	50%

### PD-L1 Expression

Absolute PD-L1 expression by tumor cells in BCCs ranges from 22% to 89.9%, while the expression by tumor-infiltrating lymphcytes (TILs) ranges from 82.0% to 94.9% (14, 15). Interestingly, Chang et al., 2017 investigated differences in PD-L1 expression in treated versus treatment-naïve BCCs (15). The cohort included 78 treated BCCs, with treatments comprising radiotherapy (n = 9), systemic chemotherapy (n = 58), and topical chemotherapy (n = 22), and 60 treatment-naïve BCCs. Topical chemotherapy included flourouracil (n=21) and imiquimod (n=1), while systemic agents included hedgehog pathway inhibitors (n=40), platinum agents (n=10), and gefitinib (n=5). Treated BCCs demonstrated greater intensity of PD-L1 expression in both tumors (32% vs 7%, P = .003) and TILs (47% vs 18%, P = .008), suggesting treatment may induce PD-L1 expression. A limitation of this study was that paired samples were not obtained from the same BCC before and after each treatment exposure. Therefore, while PD-L1 expression was associated with the above treatment modalities, the authors were unable to determine the direction of causality. However, as PD-L1 expression correlates with response to immunotherapy in other malignancies, these data imply that previously treated BCCs could possibly be more responsive to checkpoint inhibition.

In cSCCs, absolute PD-L1 expression by tumor cells ranges from 26.5% to 41% with expression by TILs reported to occur in 60% of cases (16, 17). Notably, multiple studies have suggested that high PD-L1 expression and greater intensity of expression correlate with risk of metastatic progression (17, 21). In a 2016 study by Slater and Googe, PD-L1 positivity was recorded in 20% of low grade tumors, 70% of high grade tumors, and 100% of metastases, with expression intensity increasing with grade (17). Of note, the majority of data on cSCCs derives from studies in immunocomptent patients, as compared to the subset of patients who develop cSCCs in the setting of chronic immunosuppression, especially organ transplant recipients. Accordingly, the use of ‘cSCC’ in this manuscript refers to tumors arising in the immunocompetent unless otherwise specified.

For MPyV-associated MCCs, PD-L1 expression by tumor cells and TILs has been reported at 50% and 56%, respectively (18). For non-virus associated MCCs these values are 0% and 25% (18). PD-L1 expression in MCCs may be a marker of a robust host immune response, with PD-L1 negative MCCs associated with a significantly lower overall survival (18).

### Immunogenicity: BCCs *Versus* cSCCs

Higher TMB generally predicts favorable responses to immunotherapy. However, despite BCCs and cSCCs exhibiting similar TMBs, the responses of these tumors to both immune surveillance and immunotherapy diverge significantly. While the incidence of BCC:cSCC is 4:1 in the general population, in immunosuppressed organ transplant recipients, this incidence ratio shifts to favor cSCCs, with an incidence as a high as 1:10 (22). This suggests that SCCs are more frequently recognized by and vulnerable to immune surveillance than BCCs; therefore, in immunosuppressed patients, cSCCs appear more frequently.

The relative immune privilege of BCCs remains a topic of active investigation. However, a variety of characteristics have been noted that may explain it. First, BCCs have reduced capacity for antigen presentation than cSCCs. Most cSCCs display MHC-1, but BCCs have been found to have limited to no MHC-1 expression (22). In addition, BCCs have decreased levels of transporter associated with antigen presentation (TAP-1), which may impair antigen processing prior to presentation (23). However, comparisons of TAP-1 expression between cSCCs and BCCs have not been published. BCCs also exhibit reduced numbers of invasive front, peritumoral, and intratumoral CD8+ cells compared to cSCCs (22). This may be due in part to their aforementioned reduced expression of MHC-1, as it is required for antigen recognition by CD8+ effector T cells. Furthermore, BCCs promote a more favorable local cytokine milieu than cSCCs. Both BCCs and cSCCs express high levels of Il-10, which promotes a Th2 phenotype among surrounding T cells, impairing cell-mediated toxicity (24). Compared to cSCCs, BCCs also exhibit greater expression of Th-2 cytokines IL-4 and IL-5, as well as IL-1beta and IL-6, which have been associated with more aggressive tumor behavior (25). These differences in the molecular immunogenicity of cSCCs and BCCs have implications for their respective clinical responses to immunotheray, as will be discussed below.

## Immune Checkpoint Inhibition for Cutaneous Squamous Cell Carcinoma: Existing Clinical Evidence

**Figure 2:**
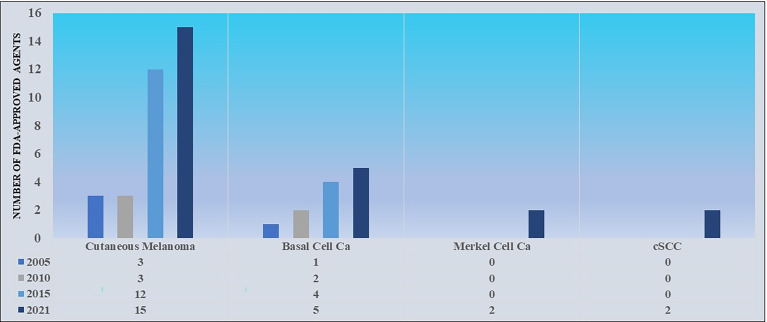
A comparison of the number of FDA-approved agents approved for the treatment of cutaneous melanoma versus NMSCs from 2005-2021. Data sourced from FDA.gov.

**Figure 3:**
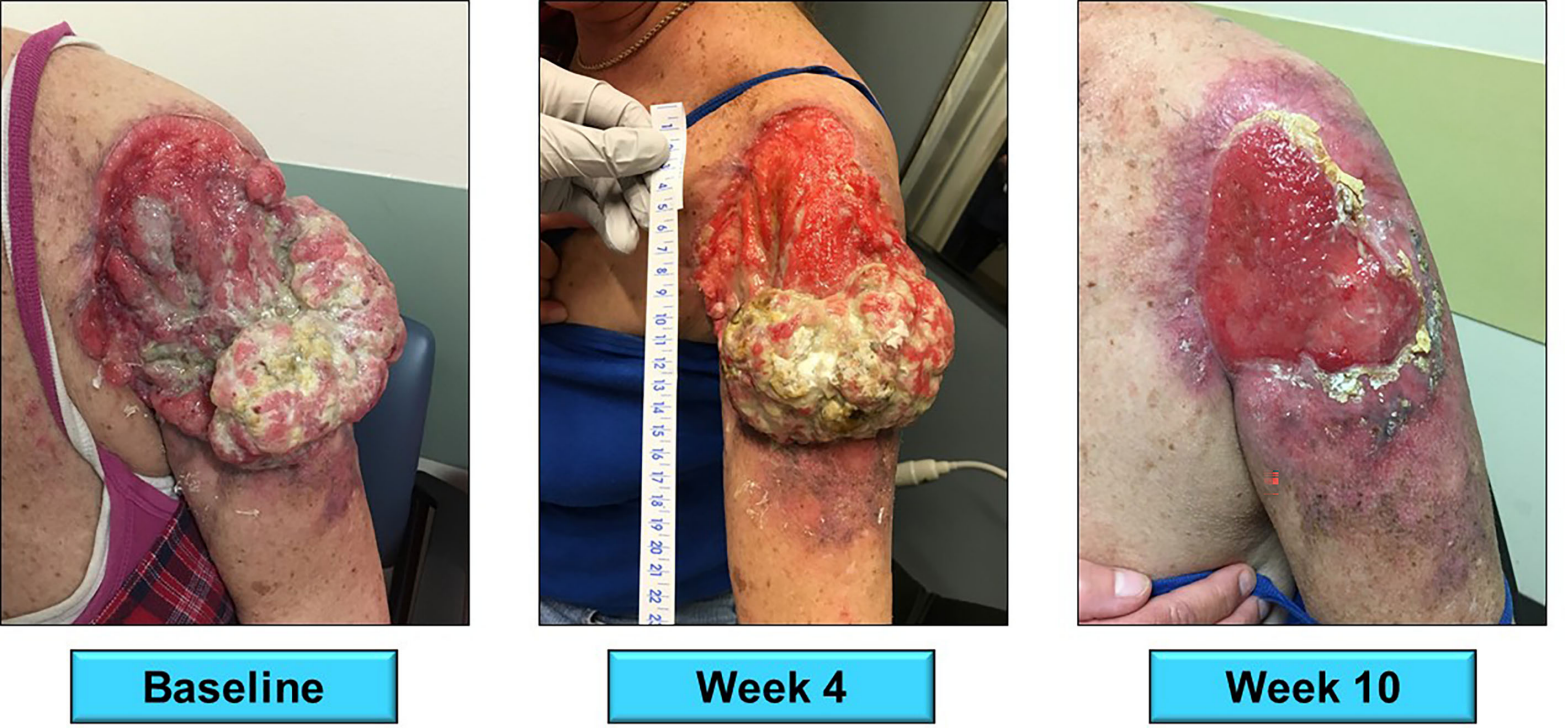
A 59 year-old female presented with locally advanced cSCC of the left upper arm. The tumor had been present for five years per patient history. She received 8 doses of nivolumab 240mg (q2 weeks) from 3/2018 to 8/2018 with complete response. Her response after 10 weeks of therapy is presented above. A subsequent radical resection was negative for residual tumor.

Immunotherapy for cSCCs has been trialed throughout the late 20^th^ and early 21^st^ centuries using interferons, interleukins, and imiquimod (26). Results were generally unimpressive, leaving providers searching for new therapies. In contrast to the treatment of cutaneous melanoma, where rapid drug development has led to a considerable array of FDA-approved therapies, the treatment of locally advanced and metastatic cSCCs has only recently seen its first, specific FDA-approved therapies (see **Figure 2** for a comparison of the number of FDA-approved agents approved in cutaneous melanoma and NMSCs from 2005-2021). The advent of checkpoint inhibition with PD-L1/PD-1 inhibitors and its use in cases of advanced cSCC, especially unresectable forms, drew attention for its potential to lead to remarkable results (see **Figure 3**). Historically, it was not until 2016 that a series of case reports lent credence to the potential of PD-L1/PD-1 inhibition to treat locally advanced and metastatic cSCC (26–28). Chang et al. described a report of an unresectable cSCC in a male in his 70s treated with an off-label trial of pembrolizumab, which led to significant tumor reduction and stable disease during the window of observation (27). Assam et al. subsequently reported a dramatic response to off-label pembrolizumab in a 67 year-old male with complete regression of an unresectable cSCC with an *MLH1* mutation (28). Later that year, Falchook et al. published the first case of a patient with metastatic cSCC treated with cemiplimab, then as part of clinical trial NCT02383212 (29).

**Figure 4:**
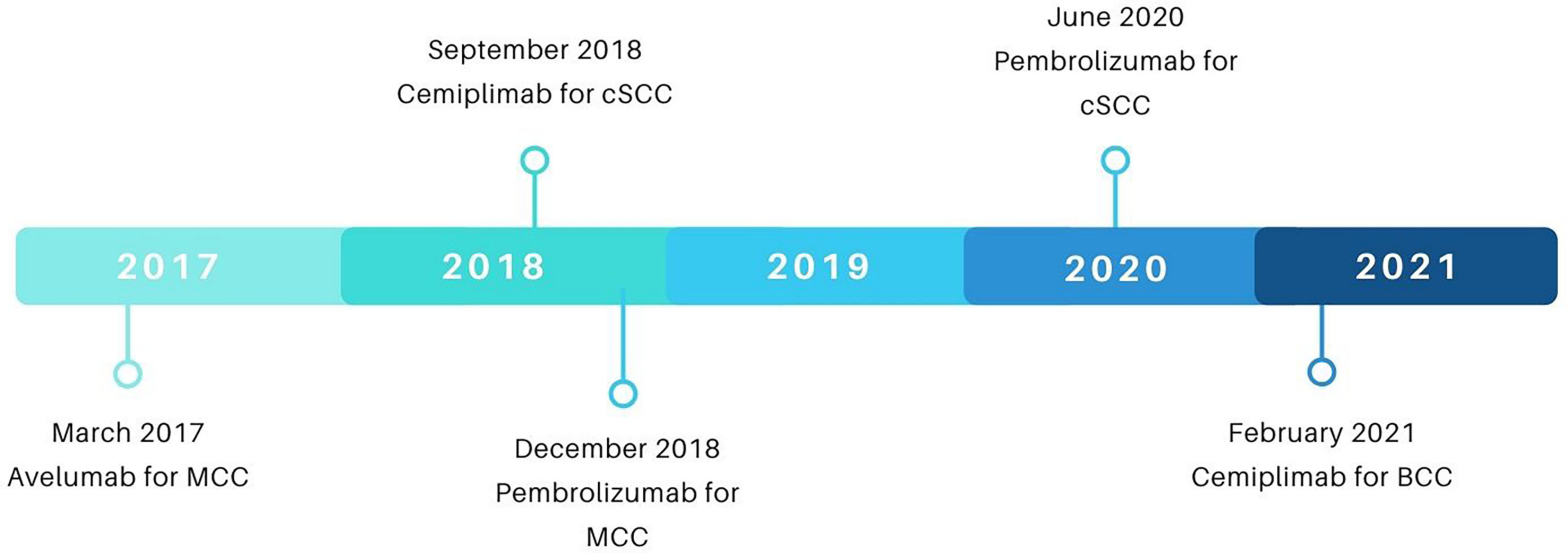
A timeline of FDA approvals of checkpoint inhibitors for NSMCs. Data sourced from FDA.gov.

In September of 2018, the FDA approved cemiplimab for metastatic and locally advanced cSCC following results from the aforementioned phase 1, open-label, multi-center, dose-finding trial with expansion cohorts (NCT02383212) as well as its follow-up phase 2 study (NCT02760498) (see **Figure 4** for a summary of FDA approvals of checkpoint inhibitors for NSMCs). In both studies, dosages were standardized at 3 mg per kilogram of body weight every 2 weeks (30). 108 patients, inclusive of locally advanced (n=33) and metastatic (n=75) disease, comprised the evaluable population (31). The ORR for both cohorts was 47%, with complete responders and partial responders representing 4% and 44% of the ORR, respectively (30, 31). Stratified ORR included 41-49% for patients with metastatic disease depending on dosage cohort and 44% for those with locally advanced disease (see **Table 2** for a summary of response kinetics associated with PD-L1 status in key trials) (32, 33). 12-month follow-up data following FDA approval demonstrated median observed time to response of 1.9 months (range: 1.7-9.1) and median progression-free survival of 18.4 months (34). As a comparison, the median overall survival in patients with cSCC treated with traditional chemotherapy alone, including EGFR inhibitors, was 15.3 months (95% CI, 10.4-21.0) overall, with 16.2 months for locally advanced cSCC and 15.3 months for metastatic cSCC (43). The most common treatment-related adverse events observed in patients underoing cemiplimab therapy included diarrhea (28.8%), fatigue (25.4%), and nausea (23.7%) (30). Immune-related adverse events of grade 3 or higher were reported in 13.6% of patients (30).

**Table 2:** Response and biomarker data in key NMSC immune checkpoint inhibition trials (32–42).

Indication	Trial	Patients	Agent	ORR	Median TTR	Median DOR	Median TMB responders (TMB NR)	ORR for PD-L1-	ORR for PD-L1+	Notes
BCCs										
laBCC	NCT03132636	84	Cemiplimab	31%	Not reported	Not reached	58.2 mut/Mb (23.5)	Not reported	Not reported	Prior HHI failure
mBCC	NCT03132636	28	Cemiplimab	21%	3.2 mo	Not reached	Not reported	Not reported	Not reported	Prior HHI failure
MCCs										
mMCC	NCT02155647	88	Avelumab	33%	Not reported	40.5 mo	Not reported	19%	37%	Prior treatment
mMCC	NCT02155647	116	Avelumab	40%	Not reported	18.2 mo	Not reported	33%	62%	Treatment-naïve
mMCC	NCT02267603	25	Pembrolizumab	56%	2.8 mo	Not reached	Not reported	57%	61%	Treatment-naïve
Advanced MCC	NCT02488759	22	Nivolumab	68%	2.0 mo	Not reached	Not reported	Not reported	Not reported	With or without prior treatment
cSCCs										
laSCC	NCT02760498	78	Cemiplimab	44%	1.9 mo	Not reached	74 mut/Mb (29)	35%	55%	With or without prior treatment
mSCC	NCT02760498	59	Cemiplimab	49%	1.9 mo	Not reached	53.2 mut/Mb (19.4)	Not reported	Not reported	3mg/kg q2w group; with or without prior treatment
mSCC	NCT02760498	56	Cemiplimab	41%	2.1 mo	Not reached	61.4 mut/Mb (13.7)	Not reported	Not reported	350 mg q3w group; with or without prior treatment
laSCC and mSCC	NCT03284424	105	Pembrolizumab	34%	1.5 mo	Not reached	Not reported	20%	33%	With or without prior treatment
laSCC and mSCC	NCT02883556	34	Pembrolizumab	39%	Not reported	Not reached	Not reported	Not reported	Not reported	Treatment-naïve

laBCC, locally advanced basal cell carcinoma; mBCC, metastatic basal cell carcinoma; laSCC, locally advanced squamous cell carcinoma; mSCC, metastatic squamous cell carcinoma; mMCC, metastatic merkel cell carcinoma; OR, Objective response rate; TTR, Time to response; DOR, Duration of response; TMB, Tumor mutational burden; NR, Non-responder; PD-L1-, PD-L1 expression <1%; PD-L1+, PD-L1 expression >=1%; mut/mb, mutations per megabase; mo, month; HHI, Hedgehog inhibitor.

Following cemiplimab’s FDA approval, investigation into the use of immunotherapy in cSCC continued with enthusiasm. In June of 2020, the FDA approved pembrolizumab for patients with recurrent or metastatic cutaneous squamous cell carcinoma. This was based on results from a phase 2 trial (NCT03284424) of 105 patients (35). The ORR was 34.3%, with 3.8% and 30.5% of patients achieving a complete response or partial response, respectively. Sub-analysis by metastatic versus locally advanced disease was not available at the time of this review. Median duration of response was not reached; however, responses ranged from 2.7 to 13.1 months at the time of review. 79.5% of responders had an ongoing response past 6 months. Median progression free survival was 6.9 months, 12-month progression free survival rate was 32.4%, and 12-month overall survival rate was 60.3%. The most common adverse events were pruritus (14.3%), asthenia (13.3%), and fatigue (12.4%). 5.7% of patients had grade 3 or above treatment-related adverse events. In line with the above data, an additional study of pembrolizumab monotherapy in patients with unresectable cSCC (NCT02883556) demonstrated an ORR of 39% (36).

Cemiplimab remains the mainstay of most clinical regimens, due to more robust data, including higher patient numbers, longer follow-up and numerically better response rates. However, despite these numerical differences, it is unclear if this difference in efficacy between cemiplimab and other PD-1 agents, such as pembrolizumab, is truly significant. Other inhibitors are under active investigation for the treatment of cSCCs, including avelumab, nivolumab, and ipilimumab. Head-to-head comparison studies have not been conducted between these various agents, but would be necessary to definitely evaluate for true differences in efficacy.

## Immune Checkpoint Inhibition for Basal Cell Carcinoma: Existing Clinical Evidence

The initial evidence for immune checkpoint inhibition activity in BCC came from limited case reports in the mid-to-late 2010s describing responses in locally advanced and metastatic disease. Mohan et al. noted that a patient undergoing treatment with ipilimumab for metastatic melanoma achieved an incidental regression of locally advanced BCC (44). Ikeda et al. reported a patient with metastatic BCC who achieved near complete remission after treatment with nivolumab (45). Lipson et al. describe a patient with BCC metastatic to the lung who achieved a durable partial response to pembrolizumab (14). Other reports further gave credence to the thesis that formal studies of immune checkpoint inhibition in BCC were warranted (46, 47).

In 2019, the first clinical trial showed immune checkpoint inhibition activity in BCC from a Phase 1/2 investigator-initiated open-label study of pembrolizumab with or without the hedgehog inhibitor vismodegib (NCT02690948) in patients with advanced BCC (48). Of the 9 patients who received pembrolizumab alone, 44% (n=4) achieved partial responses with a median (DOR) of 67.6 weeks. Among the 7 patients receiving pembrolizumab with vismodegib, 29% (n=2) achieved a partial response for a median DOR of 52.8 weeks. Among all patients, the one-year progression free survival (PFS) was 70% and the 1-year overall survival (OS) rate was 94%. The most common immune-related adverse events included dermatitis and fatigue, and one patient experienced grade 3 hyponatremia attributable to pembrolizumab (48).

In February 2021, the FDA approved cemiplimab for patients with locally advanced and metastatic BCC. The approval was based on a phase 2 trial of cemiplimab in patients who had previously failed or were intolerant to hedgehog pathway inhibition (NCT03132636). Among 84 patients with locally advanced disease who were not candidates for curative surgery or radiation therapy (RT), 6% (n=5) of patients achieved a complete response (CR) and 25% (n=21) achieved a partial response, with a median follow-up of 15.1 months (See **Table 2** for a summary of response kinetics associated with PD-L1 status in key trials). Median duration of response (DOR) was not reached, but 85% of responses were ongoing at 12 months. The most common adverse events (AE) in this cohort were fatigue, diarrhea, and pruritis, and 17% of patients discontinued treatment due to AEs (37). Among the 28 evaluable patients in the study with metastatic BCC, 21% (n=6) of patients achieved a PR with a median follow-up duration of 9.5 months. The median DOR was not reached, but the observed duration of responses ranged between 9 and 23 months. The median time to achieve a response was 3.2 months, ranging from 2.1 to 10.5 months. Median progression free survival (PFS) was 8.3 months and median overall survival (OS) was 25.7 months. The most common AEs included fatigue, diarrhea, pruritis, and constipation (38).

## Immune Checkpoint Inhibition for Merkel Cell Carcinoma: Existing Clinical Evidence

The notion of treating MCC with immune checkpoint inhibitors was first discussed in late 2011 following the approval of ipilimumab for metastatic melanoma earlier that year (49). Following the then-recent developments linking MCPyV and the immune system to MCC, Bhatia et al. suggested the use of anti-CTLA-4 antibodies such as ipilimumab as potential therapeutic strategies to counteract lymphocytic exhaustion (49). In 2013, several groups reported PD-L1 expression on MCC tumor cells and/or PD-1 expression on TILs in the tumor microenvironment (TME), strengthening the rationale for immunotherapy agents that block the PD-1/PD-L1 axis to be used in MCC treatment (18, 50, 51). In mid-2015, a phase I study of pembrolizumab included one patient with previously-untreated MCC who experienced a DOR of 56+ weeks at time of publication (52). Later that year, Mantripragada and Birnbaum published the first case report of checkpoint inhibitor use in the treatment of MCC which detailed a 42-year-old patient with refractory metastatic MCC who experienced symptomatic relief and shrinkage of heart and pancreatic metastases following four rounds of nivolumab (53).

In 2016, Kaufman et al. published the first results from a clinical trial of immune checkpoint inhibitors in MCC with Part A of the pivotal phase II JAVELIN Merkel 200 trial where they demonstrated objective responses in 32% of 88 refractory metastatic MCC patients treated with avelumab, logging 8 CRs and 20 PRs (54). Notably, 74% of responses persisted beyond one year, greatly improving on the roughly three month DOR seen in first-line chemotherapy at the time (55). FDA approval of avelumab for refractory metastatic MCC followed in March 2017. In 2018, early data from Part B of JAVELIN Merkel 200, which focused on the study of avelumab as a first-line agent in metastatic MCC, indicated a confirmed objective response in 62% of 29 patients with 83% of responders achieving a DOR of 6+ months (56). A later update in 2019 revealed a median duration of response of 18.2 months in 116 patients and median overall survival of 20.3 months, though with a decreased ORR of 39.7% (see **Table 2** for a summary of response kinetics associated with PD-L1 status in key trials) (39). Extended Part A JAVELIN Merkel 200 survival data over a median 65.1 month follow-up period was published in 2021 and revealed median overall survival of 12.6 months and overall survival rates of 30% and 26% at four and five years, respectively (57). Of note, avelumab has been recommended as first-line treatment for metastatic MCC by the NCCN since 2018 (58).

In mid-2016, Nghiem et al. published results from the KEYNOTE-017 trial, which investigated pembrolizumab in 25 advanced MCC patients without prior systemic therapy (40). Sixteen percent (n=4) of patients experienced a CR and 40% (n=10) achieved a PR for an overall objective response rate of 56%. Of note, response was observed in patients with both MCPyV+ and MCPyV- tumors and response to pembrolizumab was not found to be correlated with PD-L1 expression. The FDA granted approval to pembrolizumab in late 2018 for recurrent locally advanced or metastatic MCC ahead of the release of updated data from Nghiem et al’s KEYNOTE-017 trial, which featured an overall response rate of 56% and increased the strength for pembrolizumab as a first-line agent in advanced MCC (59).

The first significant data exploring the role of nivolumab in treating advanced MCC was presented in 2017 by Topalian et al. as part of the CheckMate358 trial. Of 22 evaluable patients, 14% (n=3) had CR and 55% (n=12) had PR for a 68% objective response rate (71% in treatment-naïve individuals and 63% in those with 1-2 prior systemic therapies) (41). Most recently in 2020, data from CheckMate358 examining nivolumab as neoadjuvant therapy before surgical resection revealed pathological CR in 17 of 36 individuals (47.2%) who underwent surgery and tumor reduction of ≥30% in 18 of 33 individuals (54.5%) of people who could be radiographically evaluated (60).

Avelumab, pembrolizumab, and nivolumab all demonstrate significant promise in the treatment of MCC; nonetheless, adverse events reported in the trials of these therapies align with previously reported adverse effects in checkpoint inhibitors. The most common adverse effects among the main MCC trials were fatigue, infusion-related reactions, diarrhea, nausea, and lab abnormalities (e.g. elevated liver enzymes) (39, 54, 57, 60). Of note, avelumab is associated with a high rate of infusion reactions, with 25% of patients receiving avelumab experiencing an infusion reaction versus less than 10% of patients receiving other immune checkpoint inhibitors (61).

## Heterogeneity of Responses

NMSCs differ in their responses to checkpoint inhibition, a fact which likely reflects the subtle differences in their immunological characteristics, as described in the section on Immunogenicity above. These distinctions are important for both future drug development as well as the establishment of clear clinical expectations during treatment.

### cSCC and BCC

The greater immunogenicity of SCCs compared to BCCs is reflected in their respective responses to immunotherapy, both in terms of overall response rate and median time to response. In patients with metastatic BCC, cemiplimab produced an overall response rate (ORR) of 21% by investigator assessment, while, in patients with metastatic cSCC, the overall response rate was 47% (30, 38). Among patients with metastatic BCC who responded to cemiplimab, the median time to achieve a response was 3.2 months, ranging from 2.1 to 10.5 months, versus 2.3 months, ranging from 1.7 to 7.3 months, for those with metastatic cSCC who responded to cemiplimab (30, 38). cSCCs appear to respond more vigorously and more quickly to immunotherapy than BCCs.

### MCPyV-Associated MCC and Non-MCPyV-Associated MCC

While non-Merkel cell polyomavirus (MCPyV)-associated MCCs display high tumor mutational burden at a median 53.9 mutations/Mb, MCPyV-associated MCCs do not. Rather, they are associated with a cohort of low-TMB MCCs with a median TMB of 1.2 mutations/Mb (see **Table 1**) (13). Despite these considerable difference in tumor neoantigen expression, response rates to checkpoint inhibition were 50% in TMB-high/UV-driven MCCs and 41% in TMB-low/MCPyV-positive tumors, a non-significant difference (p=0.63) (13). The similarity in responses between these tumor types suggests that the viral antigens in MCPyV-associated MCCs increase the immunogenicity of the respective tumor to a level equivalent to MCCs with a high mutational burden related to UV exposure, leaving them both susceptible to immunotherapy.

## Future Directions

Checkpoint inbibition in NMSCs is an area of active, ongoing investigation. **Tables 3**–**5** present a summary of current and future trials for cSCCs, BCCs, and MCCs.

**Table 3:** Active and upcoming trials in immune checkpoint inbibition for cSCC.

Identifier	Treatment Setting/Trial Phase	Immune Checkpoint Inhibitor(s) Involved	Other Involved Agent(s) including RT	Recruitment Status
**NCT02760498**	Unresectable Locally Advanced cSCC or Metastatic cSCC/Phase I	Cemiplimab	None	Recruiting
**NCT02955290**	Stage III-IV cSCC of the Head and Neck/Phase I-II	Nivolumab, Pembrolizumab	CIMAvax (EGF vaccine)	Recruiting
**NCT02964559**	Locally Advanced cSSC or Metastatic cSCC/Phase II	Pembrolizumab	None	Active, not recruiting
**NCT03082534**	Unresectable Locally Advanced cSCC/Phase II	Pembrolizumab	None	Recruiting
**NCT03284424**	Locally Advanced cSCC, Metastatic cSCC, or Recurrent cSCC/Phase II	Pembrolizumab	None	Active, not recruiting
**NCT03565783**	Stage III-IV cSCC of the Head and Neck/Phase II	Cemiplimab	None	Recruiting
**NCT03666325**	Unresectable Locally Advanced cSCC or Metastatic cSCC/Phase II	Pembrolizumab	Cetuximab	Not yet recruiting
**NCT03737721**	Unresectable cSCC/Phase II	Avelumab	RT	Recruiting
**NCT03833167**	High-Risk Locally Advanced cSCC/Phase III	Pembrolizumab	None	Recruiting
**NCT03834233**	Locally Advanced cSCC or Metastatic cSCC/Phase II	Nivolumab	None	Active, not recruiting
**NCT03889912**	Recurrent and Resectable cSCC/Phase I	Cemiplimab	None	Active, not recruiting
**NCT03944941**	Nonresectable Locally Advanced cSCC or Metastatic cSCC/Phase II	Avelumab	Cetuximab	Recruiting
**NCT03969004**	High risk cSCC/Phase III	Cemiplimab	None	Active, not recruiting
**NCT04050436**	Locally Advanced cSCC or Metastatic cSCC/Phase II	Cemiplimab	Cetuximab, RP1 (oncolytic virus)	Recruiting
**NCT04154943**	Stage II-IV (M0) cSCC/Phase II	Cemiplimab	None	Recruiting
**NCT04204837**	Stage III-IV cSCC/Phase II	Nivolumab	None	Active, not recruiting
**NCT04242173**	Unresectable Locally Recurrent cSCC or Metastatic cSCC/Phase II	Cempilimab	None	Recruiting
**NCT04315701**	Resectable High Risk Localized cSCC or Resectable Locally Recurrent cSCC or Resectable Regionally Advanced cSCC/Phase II	Cempilimab	None	Recruiting
**NCT04339062**	Locally Advanced cSCC or Metastatic cSCC in people with either prior allogeneic HSCT or renal transplant/Phase I	Cempilimab	None	Recruiting
**NCT04428671**	Resectable High Risk cSCC/Phase I	Cemiplimab	None	Recruiting
**NCT04611321**	Unresectable Locally Advanced cSCC or Metastatic cSCC/Phase I-II	IBI318 (anti-PD-1/anti-PD-L1)	None	Recruiting
**NCT04620200**	Resectable Stage III-IVa cSCC (Stage I-II cSCC if Extensive/Mutilating Surgery is Required)/Phase II	Nivolumab, Ipilimumab	None	Recruiting
**NCT04632433**	High Risk Resectable Stage III cSCC/Phase II	Cemiplimab	None	Not yet recruiting
**NCT04710498**	Resectable cSCC/Phase II	Atezolizumab	None	Not yet recruiting
**NCT04808999**	Resectable High Risk cSCC or Resectable Locooregional cSCC/Phase II	Pembrolizumab	None	Not yet recruiting
**NCT03901573**	*Locoregionally Advanced cSCC/MCC Needing Systemic Treatment or Metastatic cSCC/MCC/Phase Ib-II	Atezolizumab	NT-17 (IL-7 agonist)	Recruiting
**NCT03816332**	*Stage III-IV MCC, Unresectable MCC, Unresectable BCC, Metastatic BCC, Metastatic cSCC/Phase I	Nivolumab, Ipilimumab	Tacrolimus	Recruiting
**NCT02978625**	Advanced BCC/MCC/cSCC or Non-Refractory BCC/MCC/cSCC/Phase II	Nivolumab	TVEC	Recruiting

*Melanoma(s) are included in these trials.

**Table 4:** Active and upcoming trials in immune checkpoint inhibition for BCC.

Identifier	Treatment Setting/Trial Phase	Immune Checkpoint Inhibitor(s) Involved	Other Involved Agent(s) including RT	Recruitment Status
**NCT03132636**	Locally Advanced BCC or Metastatic BCC/Phase II	Cemiplimab	None	Active, not recruiting
**NCT03521830**	Locally Advanced BCC or Metastatic BCC/Phase II	Nivolumab, Ipilimumab, Relatlimab (anti-LAG-3)	None	Recruiting
**NCT04323202**	Locoregionally Advanced and Resectable BCC/Phase II	Pembrolizumab	None	Recruiting
**NCT04679480**	Locally Advanced BCC, Metastatic BCC, or Presence of >5 BCCs/Phase II	Cemiplimab	Sonidegib (small molecule Hedgehog pathway inhibitor)	Recruiting
**NCT03816332**	*Stage III-IV MCC, Unresectable MCC, Unresectable BCC, Metastatic BCC, Metastatic cSCC/Phase I	Nivolumab, Ipilimumab	Tacrolimus	Recruiting
**NCT02978625**	Advanced BCC/MCC/cSCC or Non-Refractory BCC/MCC/cSCC/Phase II	Nivolumab	TVEC	Recruiting

*Melanoma(s) are included in these trials.

**Table 5:** Active and upcoming trials in immune checkpoint inhibition for MCC.

Identifier	Treatment Setting/Trial Phase	Immune Checkpoint Inhibitor(s) Involved	Other Involved Agent(s) including RT	Recruitment Status
**NCT02196961**	Completely Resected MCC/Phase II	Nivolumab	None	Active, not recruiting
**NCT02584829**	Stage IV MCC/Phase I-II	Avelumab	IFN-beta, MCPyV-specific CD8+ cells, RT	Active, not recruiting
**NCT03071406**	Stage IV MCC/Phase II	Nivolumab, Ipilimumab	RT	Recruiting
**NCT03271372**	Stage III MCC/Phase III	Avelumab	None	Recruiting
**NCT03304639**	Stage III-IV MCC/Phase II/Phase II	Pembrolizumab	RT	Active, not recruiting
**NCT03599713**	Advanced/Stage IV MCC	Retifanlimab (anti-PD1)	None	Recruiting
**NCT03712605**	Completely Resected Stage I-III MCC/Phase III	Pembrolizumab	RT	Recruiting
**NCT03747484**	Nonresectable MCC or Stage IV MCC/Phase I-II	Avelumab, Pembrolizumab	FH-MCVA2TCR (Autologous MCPyV-specific T-cells)	Recruiting
**NCT03783078**	Locoregionally Advanced MCC or Stage IV MCC/Phase III	Pembrolizumab	None	Active, not recruiting
**NCT03798639**	Stage III MCC/Phase I	Nivolumab, Ipilimumab	RT	Active, not recruiting
**NCT03853317**	Stage IV MCC/Phase II	Avelumab	N-803 (IL-15 superagonist), haNK (CD16-targeted NK cells)	Recruiting
**NCT03988647**	Stage IV MCC/Phase II	Pembrolizumab	RT	Recruiting
**NCT04261855**	Stage IV MCC/Phase Ib-II	Avelumab	RT	Recruiting
**NCT04291885**	Stage I-III MCC/Phase II	Avelumab	None	Recruiting
**NCT04393753**	Stage III-IV MCC/Phase II	Avelumab	Domatinostat (HDAC inhibitor)	Recruiting
**NCT04792073**	Refractory Stage III-IV MCC/Phase II	Avelumab	RT	Recruiting
**NCT03901573**	*Locoregionally Advanced cSCC/MCC Needing Systemic Treatment or Metastatic cSCC/MCC/Phase Ib-II	Atezolizumab	NT-17 (IL-7 agonist)	Recruiting
**NCT03816332**	*Stage III-IV MCC, Unresectable MCC, Unresectable BCC, Metastatic BCC, Metastatic cSCC/Phase I	Nivolumab, Ipilimumab	Tacrolimus	Recruiting
**NCT02978625**	Advanced BCC/MCC/cSCC or Non-Refractory BCC/MCC/cSCC/Phase II	Nivolumab	TVEC	Recruiting

*Melanoma(s) are included in these trials.

### Neoadjuvant Therapy

Neoadjuvant therapy for cutaneous melanoma is currently being investigated, with recent data suggesting promising results. In a meta-analysis of six clinical trials, 33% of patients achieved a pathologic complete response (pCR) with neoadjuvant immunotherapy (43% combination and 20% monotherapy) (62). In patients with pCR, near pCR or partial pathologic response with immunotherapy, the two-year relapse free survival was 96% (62). The efficacy of neoadjuvant immunotherapy in cutaneous melanoma has inspired similar trials in NMSCs.

Numerous phase 1 and 2 trials are investigating neoadjuvant checkpoint inhibition for the treatment of recurrent or metastatic BCC and cSCC. Based on promising response rates from a recent case series, a phase 1 trial was initiated in mid-2020 to evaluate the response and recurrence rates of BCCs to neoadjuvant pembrolizumab with an additional year of adjuvant treatment after resection if required (NCT04323202). Neoadjuvant administration of checkpoint inhibitors is also an active area of clinical research for cSCCs with trials investigating neoadjuvant cemiplimab (NCT03889912, NCT04428671, NCT04632433), nivolumab (NCT04620200), atezolizumab (NCT04710498), and pembrolizumab (NCT04808999) to begin recruiting soon.

### Adjuvant Therapy

Adjuvant therapy utilizing checkpoint inhibition has demonstrated considerable efficacy in cutaneous melanoma, with studies suggesting the use of checkpoint inhibitors following resection in Stage III and IV can reduce the risk of disease relapse by 40–50% (63, 64).Due to encouraging results from initial studies of adjuvant therapy, current trials are investigating head-to-head comparisons of checkpoint inhibitors, combination therapy, and the use of adjuvant therapy in earlier stages of disease (2). The success of adjuvant therapy in cutaneous melanoma has led to its investigation in the treatment of NMSCs as well.

The use of checkpoint inbibition as adjuvant therapy for advanced NMSCs is a current focus of numerous upcoming and ongoing studies. Notable trials include the use of adjuvant pembrolizumab after resection in BCCs (NCT04323202), adjuvant nivolumab following complete MCC resection (NCT02196961), pembrolizumab following surgery and radiotherapy for cSCCs (NCT03833167), and cemibilimab following both surgery and radiotherapy (NCT03969004) as well as surgery alone (NCT04428671) for cSCCs.

## Improving the Efficacy of Checkpoint Inhibition

### Hedgehog Inhibition (BCC)

While the aforementioned investigator-initiated open-label study of pembrolizumab with or without hedgehog inhibition in advanced BCC did not find a difference in response between the single agent arm and the dual treatment arm, this approach is still undergoing clinical investigation given strong pre-clinical evidence that implicates hedgehog signaling in promoting an immuno-suppressive tumor microenvironment (65). HHI in BCC increases chemokines involved in T cell recruitment and influx of T cells, suggesting a potential for synergy between HHI and checkpoint inhibition in advanced BCC patients (66). To this end, a phase 2 trial is investigating cemiplimab in combination with pulsed sonidegib for patients with advanced BCC (NCT04679480).

### Cetuximab (*cSCC)*

Cetuximab is an EGFR-inhibitor approved for multiple indications associated with squamous cell carcinoma of the head and neck, including concomitant administration with platinum-based agents and radiotherapy as well as monotherapy in cases unresponse to platinum-based therapy. Recent studies have suggested the potential of cetuximab to treat unresectable cSCC, and numerous trials are now investigating the efficacy of combination therapy with cetuximab and various checkpoint inhibitors, including pembrolizumab (NCT03082534, NCT03666325) and avelumab (NCT03944941). An abstract at the 2021 ASCO meeting suggested ceteuximab may have a role in the treatment of patients immediately after progression on immunotherapy. In a small cohort study, patients who were initiated on cetuximab immediately following immunotherapy failure experienced an ORR of 54%, with 1 complete and 6 partial responses (67).

### HDACis (MCC)

Domatinostat is a selective class I histone deacetylase inhibitor, which functions to upregulate the expression of cancer germline antigens and MHC class I/II molecules, among other modifications in the tumor microenvironment, boosting the innate immune response (22). Domatinostat is currently being tested alongside avelumab in a trial for patients with MCC refractory to previous immune checkpoint therapy (NCT04393753).

### Radiation (MCC, BCC, cSCC)

The use of radiation in conjunction with checkpoint inhibitor therapy remains an area of active investigation. In addition to its role in directly killing tumor cells, radiotherapy has shown further potential benefit in cancer care through auxiliary means that include modulation of the tumor microenvironment, increased tumor-associated antigen expression, increased cytokine release, and stimulation and proliferation of immune cells such as CD8+ cytotoxic T-cells (68). The abscopal effect, which describes the regression of a tumor or tumors distant from the site of local radiotherapy, is believed to reflect the immune-sensitizing effect of radiotherapy and has been observed in cases of cSCC and MCC (69–71). Greater understanding of these effects has underscored the hypothesis of a synergy between radiotherapy and immunotherapies in cancer. This idea has resulted in several ongoing trials in MCC, BCC, and cSCC aimed at determining the efficacy of radiotherapy in conjunction with various checkpoint inhibitors.

### Dual Checkpoint Blockade (MCC, BCC, cSCC)

Given the success of dual immune checkpoint inhibition in various solid tumors, a phase 2 clinical trial in locally advanced and metastatic BCC patients is investigating the use of nivolumab in combination with ipilimumab or relatlimab, an investigational monoclonal antibody that blocks the immune checkpoint receptor LAG-3 (NCT03521830). Similarly, a phase 2 trial is underway examining the response rates of advanced cSCC to IBI318, an anti-PD-1/PD-L1 bispecific antibody (NCT04611321). Though avelumab has become the de-facto neoadjuvant therapy in metastatic MCC, cases of MCC refractory to initial anti-PD-L1 monotherapy have been documented. In the specific case of avelumab-refractory MCC, case reports have suggested a nivolumab + ipilimumab regimen may overcome this resistance with documented durability of response (72–74). This regimen is currently being assessed with and without stereotactic radiation therapy for treatment of avelumab-resistant metastatic MCC (NCT03071406).

### Direct Comparisons

Currently, there are no current or future studies assessing head-to-head efficacy of different immune checkpoint inhibitors across NMSCs. While certain checkpoint inhibitors, such as cempibilmab for cSCC and avelumab for MCC, are used more often in the clinical setting, it remains unknown if there are significant inter-class differences.

### Other Immunotherapies

Other novel immunomodulatory agents are being investigated as concomitant therapies to boost the efficacy of immune checkpoint inhibition in the treatment of NMSCs. Oncolytic viruses are an active area of research. A phase 2 study of talimogene laherparepvec, an oncolytic herpesvirus, in combination with nivolumab for the treatment of cSCCs, BCCs, and MCCs (NCT02978625) is ongoing. In addition, cemiplimab in combination with RP1, an oncolytic herpesvirus that encodes a fusogenic GALV-GP R-protein and GM-CSF, is being studied for the treatment of advanced cSCC (NCT04050436). An additional trial of tumor antigen vaccination with recombinant Human EGF-rP64K/Montanide ISA 51 in addition to nivolumab or pembrolizumab is in progress (NCT02955290). The administration of exogenous cytokines is also under investigation, with a study of NT-17, an IL-7 agonist, in combination with atezolizumab for the treamtent of advanced MCC and cSCC (NCT03901573). Several trials of MCC therapy involve the administration of recombinant immune cells. One current trial examines a treatment of avelumab combined with CD-16 targeted NK cells (haNK) and a novel IL-15 superagonist (N-803) in patients with MCC refractory to a first-line checkpoint inhibitor (NCT03853317). An additional trial for patients with unresectable or metastatic MCC involves the co-administration of a checkpoint inhibitor with autologous T-cells that have been genetically engineered to recognize and target MCPyV (NCT03747484).

### Future Biomarkers

Further advancement in the field of immunotherapy will depend on the expanded study of biomarkers that can serve as predictors of response and resistance to checkpoint inhibition. While TMB is known to correlate with response to PD-1 blockade, it alone does not fully predict outcomes, as some non-responders have high TMB. Therefore, identifying other factors that can influence the efficacy of immune checkpoint inhibition will enable tailored treatment. Such factors that require further investigation include known biomarkers, such as PD-L1 expression and infiltrating T cells, as well as genomic studies. In one recent example, non-amplification short variant mutations in PD-L1, were identified in 1.6% of cSCCs, potentially heralding resistance to checkpoint inhibition (75).

### Use in Solid Organ Transplantation

A current critical question in the field of immunoncoology is the appropriate use of checkpoint inhibition in the setting of solid organ transplantation. Transplant recipients carry a greatly increased risk of developing cancer, especially NMSCs (22, 76–78). For example, in kidney transplant patients, cSCCs represent 70% of all malignancies post-transplant and are estimated to affect over 50% of kidney transplant recipients (78). Checkpoint inhibitors have been used safely to address advanced disease in transplant patients (76–78). However, the risk of rejection stands between 25-50% according to recent reports (76–78). Therefore, novel ways to maintain the efficacy of checkpoint inhibition and minimize the risk of rejection are required.

## Conclusion

NMSCs represent a significant global health burden that is set to grow ever larger with time, as medical advances permit both a rising average life expectancy and, associatively, an increased risk for NMSC development. Breakthroughs in immunotherapy first touted in the treatment of melanoma have now shown promising data in the treatment of advanced NMSCs, where previously few to no effective therapies were available. The immunogenicity of NMSCs makes them an attractive target for immunotherapy, and, accordingly, clinical trials in this space are being initiated at a rapid pace. Immune checkpoint inhibition has begun to demonstrate clinical efficacy in treating NMSCs of all subtypes. Future studies will further define the array of checkpoint inhibitors that offer maximal efficacy as well as the crucial concomitant therapies necessary to optimize their therapeutic potential.

## Author Contributions

Conceptualization, CS, AD, JG, AK, LG, and RC. Methodology, CS, AD, JG, and AK. Writing—original draft preparation, CS, AD, JG, AK, YS, and AC. Writing—review and editing, CS, AD, JG, AK, YS, AC, LG, and RC. Tables and figures, CS, AD, JG, and AK. Supervision, YS, AC, LG, and RC. Project administration, CS. All authors contributed to the article and approved the submitted version.

## Conflict of Interest

The authors declare that the research was conducted in the absence of any commercial or financial relationships that could be construed as a potential conflict of interest.

## Publisher’s Note

All claims expressed in this article are solely those of the authors and do not necessarily represent those of their affiliated organizations, or those of the publisher, the editors and the reviewers. Any product that may be evaluated in this article, or claim that may be made by its manufacturer, is not guaranteed or endorsed by the publisher.
